# Interactions of Mycotoxin Alternariol with Cyclodextrins and Its Removal from Aqueous Solution by Beta-Cyclodextrin Bead Polymer

**DOI:** 10.3390/biom9090428

**Published:** 2019-08-30

**Authors:** Eszter Fliszár-Nyúl, Beáta Lemli, Sándor Kunsági-Máté, Lajos Szente, Miklós Poór

**Affiliations:** 1 Department of Pharmacology, Faculty of Pharmacy, University of Pécs, H-7642 Pécs, Hungary; 2 János Szentágothai Research Centre, University of Pécs, H-7642 Pécs, Hungary; 3 Institute of Organic and Medicinal Chemistry, Medical School, University of Pécs, H-7624 Pécs, Hungary; 4 Cyclolab Cyclodextrin Research & Development Laboratory, Ltd., H-1097 Budapest, Hungary

**Keywords:** alternariol, cyclodextrin, host-guest complexes, fluorescence spectroscopy, fluorescence enhancement, cyclodextrin polymers, mycotoxin binder, toxin removal

## Abstract

Alternariol is an *Alternaria* mycotoxin that appears in fruits, tomatoes, oilseeds, and corresponding products. Chronic exposure to it can induce carcinogenic and xenoestrogenic effects. Cyclodextrins (CDs) are ring-shaped molecules built up by glucose units, which form host–guest type complexes with some mycotoxins. Furthermore, insoluble CD polymers seem suitable for the extraction/removal of mycotoxins from aqueous solutions. In this study, the interactions of alternariol with β- and γ-CDs were tested by employing fluorescence spectroscopic and modeling studies. Moreover, the removal of alternariol from aqueous solutions by insoluble β-CD bead polymer (BBP) was examined. Our major observations/conclusions are the following: (1) CDs strongly increased the fluorescence of alternariol, the strongest enhancement was induced by the native γ-CD at pH 7.4. (2) Alternariol formed the most stable complexes with the native γ-CD (log*K* = 3.2) and the quaternary ammonium derivatives (log*K* = 3.4–3.6) at acidic/physiological pH and at pH 10.0, respectively. (3) BBP effectively removed alternariol from aqueous solution. (4) The alternariol-binding ability of β-CD polymers was significantly higher than was expected based on their β-CD content. (5) CD technology seems a promising tool to improve the fluorescence detection of alternariol and/or to develop new mycotoxin binders to decrease alternariol exposure.

## 1. Introduction

**Figure 1:**
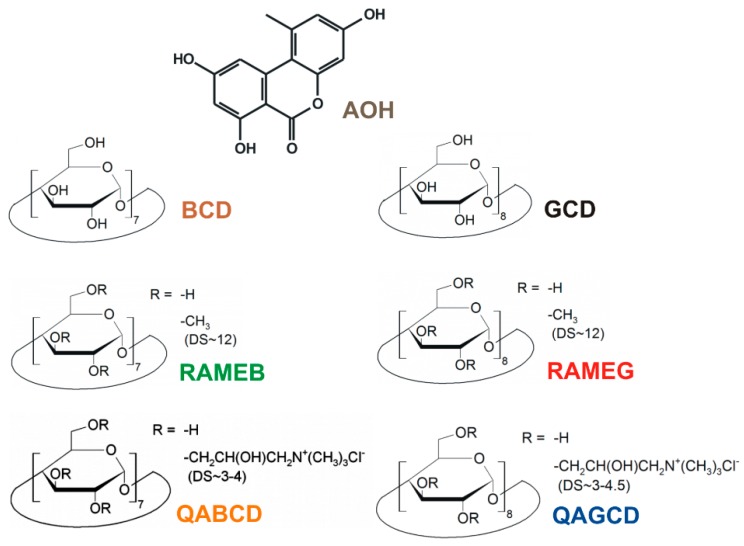
Chemical structures of alternariol (AOH) as well as native and chemically modified β- and γ-cyclodextrins (BCD, β-cyclodextrin; RAMEB, randomly methylated β-cyclodextrin; QABCD, (2-hydroxy-3-N,N,N-trimethylamino)propyl-β-cyclodextrin; GCD, γ-cyclodextrin; RAMEG, randomly methylated γ-cyclodextrin; QAGCD, (2-hydroxy-3-N,N,N-trimethylamino)propyl-γ-cyclodextrin; DS: average degree of substitution per CD ring).

Contamination of food and feed with mycotoxins has been an emerging problem worldwide. Alternariol (AOH) is a dibenzo-α-pyrone mycotoxin (Figure 1) produced by the phytopathogenic *Alternaria* fungi. Cereals, tomatoes, grapes as well as other soft-skinned fruits and vegetables are particularly susceptible for *Alternaria* infection [1]. Furthermore, AOH occurs as a contaminant in the corresponding processed products (e.g., wine and tomato juice) [2,3,4]. The concentration of AOH in different foods and beverages is in the 0.7 to 41.6 μg/L (2.7 to 161.1 nM) range [5]. However, the extremely contaminated oilseeds can contain even 1000 μg/kg concentration of AOH [6]. The high thermal stability of *Alternaria* toxins makes their removal from foodstuffs difficult [6,7]. The AOH content of different foodstuffs and beverages is generally examined by HPLC (high performance liquid chromatography) linked to FLD (fluorescence detector), MS (mass spectrometer), or DAD (diode array detector), and stable isotope dilution assay (SIDA) methods [8,9,10,11]. Since AOH exerts fluorescence in aqueous solution, its molecular interactions can be effectively investigated using fluorescence spectroscopy [12].

Cyclodextrins (CDs) are ring-shaped oligosaccharides built up from six (α-CDs), seven (β-CDs), or eight (γ-CDs) glucopyranose units, linked through α-(1,4) bonds [13]. The hydrophilic outer part of CDs provides excellent aqueous solubility, while their nonpolar internal cavity can accommodate lipophilic compounds [14]. The chemical modification of CDs affects their aqueous solubility and the stability of their host–guest type complexes, which properties are also influenced by the type and the degree of substitution [15,16]. CDs are extensively used by pharmaceutical [15,17,18], cosmetic [19], and food industries [20,21]. Their applications include solubilization and stabilization (protection against light and oxidation) of compounds, improvement of the oral bioavailability of some drugs [18,22,23], and CDs can suppress unpleasant odors and/or tastes [20]. Furthermore, CDs are employed in environmental protection [24,25] and are also useful molecules in analytics [26,27].

Native and chemically modified CDs can form stable complexes with several mycotoxins, including aflatoxins, citrinin, ochratoxin A, and zearalenone [28,29,30,31]. The complex formation can strongly increase the fluorescence of some mycotoxins, which makes possible their more sensitive fluorescence detection [29,31,32,33]. Furthermore, CD technology may be suitable for the development of new mycotoxin binders. Previous studies demonstrated that patulin, ochratoxin A, and zearalenone can be effectively removed/extracted from aqueous solutions (or even from beverages) with β-CD polymers [34,35,36,37].

In this study, the interactions of AOH with native and chemically modified (methyl and quaternary ammonium derivatives) β- and γ-CDs (Figure 1) were examined employing fluorescence spectroscopy and molecular modeling. CD-induced enhancement in the fluorescence of AOH and the stability of AOH-CD complexes were evaluated under different environmental conditions. Furthermore, the removal of AOH from aqueous solution by insoluble β-CD bead polymer (BBP) was also tested. Our results demonstrate that CDs strongly increase the fluorescence of AOH during the formation of stable host-guest type complexes; moreover, BBP can effectively remove AOH from aqueous solution.

## 2. Materials and Methods

### 2.1. Reagents

Alternariol (AOH) was purchased from Cfm Oskar Tropitzsch GmbH (Marktredwitz, Germany). Native and chemically modified CDs, including β-CD (BCD), γ-CD (GCD), randomly methylated β-CD (RAMEB), randomly methylated γ-CD (RAMEG), (2-hydroxy-3-N,N,N-trimethylamino)propyl-β-CD (or quaternary ammonium β-CD, QABCD), (2-hydroxy-3-N,N,N-trimethylamino)propyl-γ-CD (or quaternary ammonium γ-CD, QAGCD), soluble β-CD polymer (cross-linked with epichlorohydrin; BCD content: 70 m/m%), and insoluble β-CD bead polymer (BBP; CD-epichlorohydrin cross-linked bead polymer; BCD content: 50 m/m%) were obtained from CycloLab Cyclodextrin Research and Development Laboratory, Ltd. (Budapest, Hungary). HPLC-grade acetonitrile and methanol were purchased from VWR (Budapest, Hungary). Stock solution of AOH (5000 μM) was prepared in dimethyl sulfoxide (DMSO, spectroscopic grade; Fluka, NJ, USA) and was stored at −20 °C.

### 2.2. Spectroscopic Studies

Fluorescence spectroscopic measurements were carried out using a Hitachi F-4500 fluorescence spectrophotometer (Tokyo, Japan) in the presence of air, at +25 °C. Samples with AOH (5 μM = 1.29 mg/L) and increasing amounts of CDs (final concentrations: 0.0, 0.25, 0.5, 1.0, 2.0, 3.0, 4.0, 5.0, 7.0, and 10.0 mM) were prepared in 50 mM sodium acetate (pH 5.0), sodium phosphate (pH 7.4), and sodium borate buffers (pH 10.0). Since the aqueous solubility of BCD is lower vs. other CDs [38], the concentrations of BCD were the following: 0.0, 0.5, 0.75, 1.0, 1.25, 1.5, and 2.0 mM. Fluorescence emission spectra were recorded using 345 nm excitation wavelength, and the changes in the fluorescence emission of AOH was evaluated at 460 nm.

Binding constants of AOH-CD complexes were determined employing the graphical application of the following Benesi-Hildebrand equation [16,29,37]:

(1) $$\frac{I_{0}}{\left(I-I_{0}\right)}=\frac{1}{A}+\frac{1}{A\times K\times {\left[CD\right]}^{n}}$$

where *K* denotes the binding constant (unit: L/mol), *I_0_* and *I* are the fluorescence intensity (λ_ex_ = 345 nm, λ_em_ = 460 nm) of AOH without and with CDs, *[CD]* is the concentration of the host molecule (unit: mol/L), *A* is a constant, and *n* is the number of binding sites.

To confirm the determined binding constants and binding stoichiometry of AOH-CD complexes, some experimental data were also evaluated applying the Scatchard equation (for the 1:1 complex formation) [39,40]:

(2) $$\frac{\left(I-I_{0}\right)}{\left[CD\right]}=\left(I_{AOH-CD}-I_{0}\right)\times K-\left(I-I_{0}\right)\times K$$

where *I_AOH-CD_* is the fluorescence intensity when total AOH has been complexed with CDs.

### 2.3. Modeling Studies

Molecular modeling studies have been performed at semi-empirical AM1 level using HyperChem 8 code [41,42]. After geometry optimization at AM1 level, the vibrational-rotational analyses were performed in harmonic approximation. Then, the enthalpy change of the complex formation was considered as the energy change calculated by subtracting the total energies of the reactants from the total energies of the products. Similarly, the entropy changes were calculated by subtracting the entropy terms of the reactants from the entropy terms of the products. To consider the overall effect of the entropy changes, the different terms of the entropy contents of all species were calculated applying the Boltzmann-statistics. For example, after calculating the vibrational frequencies using the harmonic approximation, the entropy was determined on the common way using the following HyperChem code [41,42]:

(3) $$S_{vib}=R\underset{i}{\sum }\left\{\frac{hv_{i}/kT}{e^{\left(\frac{hv_{i}}{kT}\right)}-1}-ln\left[1-e^{\left(-\frac{hv_{i}}{kT}\right)}\right]\right\}$$

where *ν_i_* is the frequency of vibration and *T* is the temperature (298.16 K). The stability constants were determined from the Gibbs free energy changes associated to the complex formation at 298 K. A neutral aqueous environment was considered by the TIP3P solvation model implemented in HyperChem code (HyperChem, Hypercube Inc. 2007).

Cyclodextrin derivatives (QABCD, RAMEB, QAGCD, and RAMEG) were considered as charged species of the native molecules. Accordingly, the electron releasing property of the methyl groups were considered as negatively charged native BCD or GCD molecules, while the electron withdrawing character of quaternary ammonium moieties were considered as the positively charged native BCD or GCD.

### 2.4. Extraction of AOH from Aqueous Solution with Insoluble β-Cyclodextrin Bead Polymer

The removal of AOH by BBP was tested in four buffers (50 mM sodium phosphate, pH 3.0; 50 mM sodium acetate, pH 5.0; 50 mM sodium phosphate, pH 7.4; and 50 mM sodium borate, pH 10.0). AOH (2 μM = 516.5 μg/L, 1.5 mL) was incubated in the presence of 0.0, 1.0, 2.5, 5.0, 10.0, and 20.0 mg BBP in a thermomixer (1000 rpm, 30 min, 25 °C). The insoluble beads were sedimented by pulse centrifugation (4000 *g*, 3 sec, at room temperature), and then a 500 μL aliquot of the supernatant was diluted 1.5-fold with acetonitrile (before dilution, the samples at pH 10.0 were acidified with 6.5 μL of 3 mM perchloric acid). AOH contents of these samples were quantified by HPLC (see in Section 2.5).

Using the same experimental conditions, increasing concentrations of AOH (0.2, 0.5, 1.0, 2.5, 5.0, 7.5, 10.0, 12.5, and 15.0 μM in 1.5 mL buffer) were added to BBP (2.5 mg). Then, the AOH content of supernatants was determined. Using these data, the interaction of AOH with BBP was evaluated based on the Langmuir and Freundlich sorption isotherms [36,37]. The Langmuir equation is expressed as

(4) $$q_{e}=\frac{Q_{0}\times K_{L}\times C_{e}}{\left(1+K_{L}\times C_{e}\right)}$$

where *q_e_* is the amount of bound AOH (mg) by BBP (g), while *C_e_* is the amount of unbound AOH (mg) in the solution at equilibrium. *Q_0_* is the calculated maximum amount of AOH (mg) bound per g of BBP, and *K_L_* denotes the Langmuir equilibrium constant (L/mg). The Freundlich equation is described as

(5) $$q_{e}=K_{F}\times C_{e}^{1/n}$$

where *K*_F_ is the Freundlich constant (unit: (mg/g)(L/mg)^1/n^), while *n* is the heterogeneity index.

### 2.5. HPLC Analyses

The AOH content of samples was determined applying a HPLC system (Jasco; Tokyo, Japan) built up by a binary pump (PU-4180), an autosampler (AS-4050), and a FP-920 fluorescence detector. Samples (with 20 μL injected volume) were driven through a NovaPak C18 (4.0 × 3.0 mm) guard cartridge linked to a NovaPak C18 (150 × 3.9 mm, 4.0 μm) analytical column. The mobile phase was 1 mM orthophosphoric acid (pH 3) and acetonitrile (60:40 *v*/*v*%). The isocratic elution was performed with 1.0 mL/min flow rate at room temperature, then AOH was detected at 455 nm (λ_ex_ = 345 nm). Chromatographic data were evaluated using ChromNAV software (Version 2).

### 2.6. Comparison of the Interaction of Alternariol with β-Cyclodextrin, Soluble β-Cyclodextrin Polymer, and Insoluble β-Cyclodextrin Bead Polymer at pH 3

According to the manufacturer’s description, the soluble BCD polymer and the BBP contain approximately 70 and 50 m/m% BCD, respectively. Therefore, we can calculate the molar concentrations of BCD which are contained by the applied amounts of soluble BCD polymer and BBP. In the following experiments, increasing amounts of BCD (final concentrations: 0, 0.25, 0.5, 1.0, 1.5, 2.0, and 2.5 mM) or soluble BCD polymer (final concentrations were equivalent to 0, 0.14, 0.35, 0.7, 1.4, 2.1, and 2.8 mM of BCD) were added to AOH (2 μM) in sodium phosphate buffer (50 mM, pH 3.0). Then, the changes in the emission signal of AOH were recorded (λ_ex_ = 345 nm, λ_em_ = 460 nm). According to these fluorescence data, the log*K* values were calculated employing the Benesi-Hildebrand equation (Equation (1)), assuming 1:1 stoichiometry of the complex formation.

Furthermore, based on the bound fraction of AOH in the presence of BBP (see details in 2.4 and 2.5), the log*K* values regarding AOH-BBP interaction was also calculated assuming 1:1 stoichiometry.

(6) AOH+CD↔AOHBCD

(7) $$K=\frac{\left[AOHBCD\right]}{\left[AOH\right]\times \left[BCD\right]}$$

where *AOHBCD* denotes the 1:1 stoichiometry complex of AOH with BCD, while *[AOH]*, *[BCD]*, and *[AOHBCD]* are the molar concentrations of unbound AOH, unbound BCD, and AOH-BCD complex, respectively.

### 2.7. Statistical Analyses

Data demonstrate mean ± standard error of the mean (SEM) derived from at least three independent experiments. Statistical significance was established based on the One-Way ANOVA test (*p* < 0.01) using the IBM SPSS Statistics software (Version 21; New York, NY, USA).

## 3. Results

### 3.1. Effect of the Environmental pH on the Fluorescence Spectrum of Alternariol

**Figure 2:**
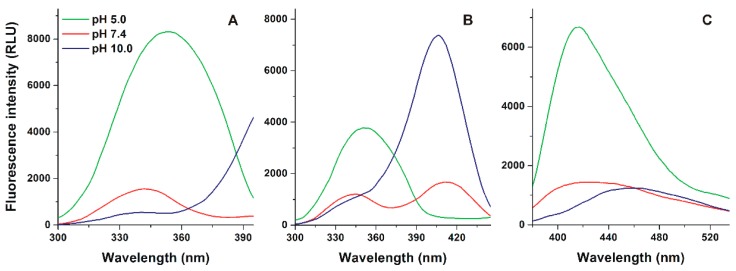
Fluorescence excitation (**A**: λ_em_ = 417 nm; **B**: λ_em_ = 465 nm) and emission (**C**: λ_ex_ = 345 nm) spectra of AOH (50 μM = 12.9 mg/L) in sodium acetate (50 mM, pH 5.0), sodium phosphate (50 mM, pH 7.4), and sodium borate (50 mM, pH 10.0) buffers (ex slit: 10 nm, em slit: 10 nm; RLU, relative light unit).

First, the fluorescence excitation spectra of AOH were recorded under acidic (pH 5.0), physiological (pH 7.4), and alkaline (pH 10.0) conditions, using 417 and 465 nm emission wavelengths. Under acidic circumstances, the excitation maximum of AOH appeared approximately at 350 nm. Then, with the elevation of the pH, a slight blue shift (350→340 nm) and the decreased intensity of this peak was observed (Figure 2A). Furthermore, at pH 7.4, a second excitation peak appeared at 410 nm, which became highly dominant at pH 10.0 (Figure 2B).

Emission spectra of AOH were recorded using 345 nm excitation wavelength. At pH 5.0, the emission maximum of AOH appeared at 417 nm, while the red shift of the emission spectrum was observed with the elevation of pH, showing emission maxima at 425 and 465 nm at pH 7.4 and 10.0, respectively (Figure 2C). Using 410 nm excitation wavelength, a large emission peak was noticed approximately at 450 nm, while this emission peak was significantly lower and missing at pH 7.4 and 5.0, respectively (Figure S1).

### 3.2. Effects of Cyclodextrins on the Fluorescence Spectrum of Alternariol

**Figure 3:**
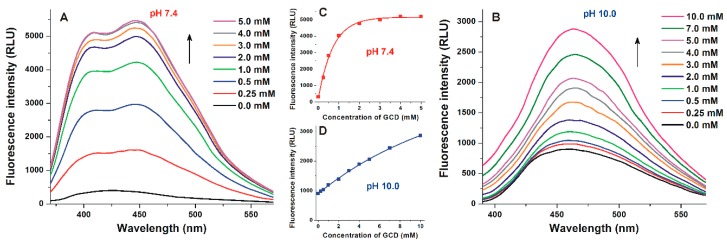
Fluorescence emission spectra of AOH (5 μM = 1.29 mg/L) in the presence of increasing concentrations of GCD (0-10 mM) in sodium phosphate (pH 7.4; **A**) and sodium borate (pH 10.0; **B**) buffers (λ_ex_ = 345 nm). GCD-induced increase in the emission signal of AOH (λ_ex_ = 345 nm, λ_em_ = 460 nm) in phosphate (pH 7.4; **C**) and borate (pH 10.0; **D**) buffers (ex slits: 10 nm at both pH; em slits: 10 and 20 nm at pH 7.4 and 10.0, respectively; RLU, relative light unit).

To characterize the interactions of AOH with CDs (native, randomly methylated, and quaternary ammonium β- and γ-CDs), fluorescence emission spectra of AOH were recorded in the presence of increasing CD concentrations using a 345 nm excitation wavelength. In the presence of CDs, a significant red shift in the emission spectrum of AOH was observed, and the AOH-CD complexes showed their emission maxima around 450–470 nm depending on the CD and the buffer used (Figure 3). Therefore, we selected 460 nm for the following comparison and evaluations (e.g., calculation of binding constants). At acidic and physiological pH, noticeably two emission peaks can be distinguished in the presence of CDs (peak 1 around 410 nm and peak 2 around 450 nm), as it is demonstrated in Figure 3 regarding GCD. This shape of the emission spectra was same in the presence of uncharged CDs (BCD, RAMEB, GCD, and RAMEG) at pH 5.0 and 7.4 (Figure 3A). However, at pH 10.0, peak 1 seems to be disappeared and a further red shift of the emission maximum was observed (Figure 3B).

**Figure 4:**
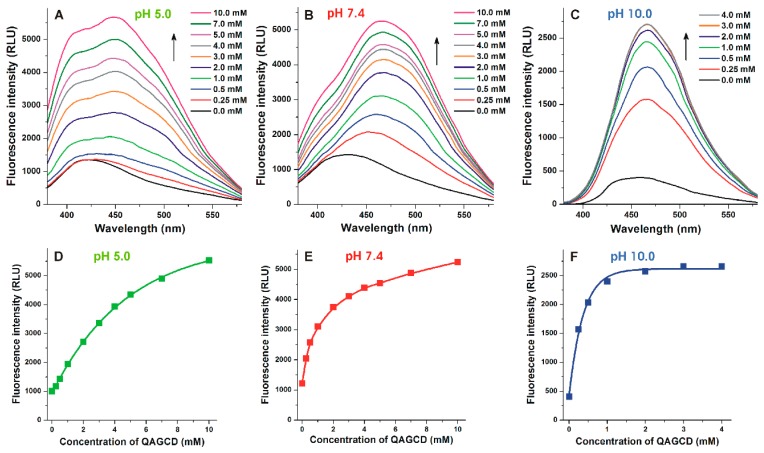
Fluorescence emission spectra of AOH (5 μM) in the presence of increasing concentrations of QAGCD (0–10 mM) in sodium acetate (pH 5.0; **A**), sodium phosphate (pH 7.4; **B**), and sodium borate (pH 10.0; **C**) buffers (λ_ex_ = 345 nm). QAGCD-induced increase in the emission signal of AOH (λ_ex_ = 345 nm, λ_em_ = 460 nm) in acetate (pH 5.0; **D**), phosphate (pH 7.4; **E**), and borate (pH 10.0; **F**) buffers (ex slits: 10 nm at each pH; em slits: 20, 20, and 10 nm at pH 5.0, 7.4, and 10.0, respectively; RLU, relative light unit).

The emission spectra of AOH in the presence of cationic CDs (tetraalkylammonium salts, QABCD and QAGCD) behaved similar to the uncharged CDs at pH 5.0 (Figure 4A) and 10.0 (Figure 4C), while the strong decrease of peak 1 vs. peak 2 was noticed even at pH 7.4 (Figure 4B).

A further typical difference between uncharged and cationic CDs is also demonstrated using GCD and QAGCD as examples. At acidic and physiological pH, GCD induced a strong increase in the fluorescence of AOH even at 0.25 mM concentration, and the emission signal of AOH reached its maximum in the presence of 4 mM GCD (Figure 3A,C). However, at pH 10.0, the emission signal of AOH only slightly increased by low GCD concentrations (0.25 and 0.5 mM) and strongly raised (as well as did not reach its plateau) even in the presence of 10 mM GCD (Figure 3B,D). In contrast, increasing concentrations of QAGCD induced a less steep, gradual increase in the fluorescence of AOH at pH 5.0 (0.25 and 0.5 mM concentrations of QAGCD produced only slight effects, and the fluorescence signal did not reach a maximum even in the presence of 10 mM QAGCD) (Figure 4A,D). At pH 7.4, a steeper elevation in fluorescence can be observed compared to the acidic environment (Figure 4B,E). Furthermore, at pH 10.0, QAGCD induced a spectacular increase in the fluorescence of AOH even at low concentration (e.g., 0.25 mM), and the emission signal of the mycotoxin reached its maximum even in the presence of 3 mM QAGCD (Figure 4C,F).

**Figure 5:**
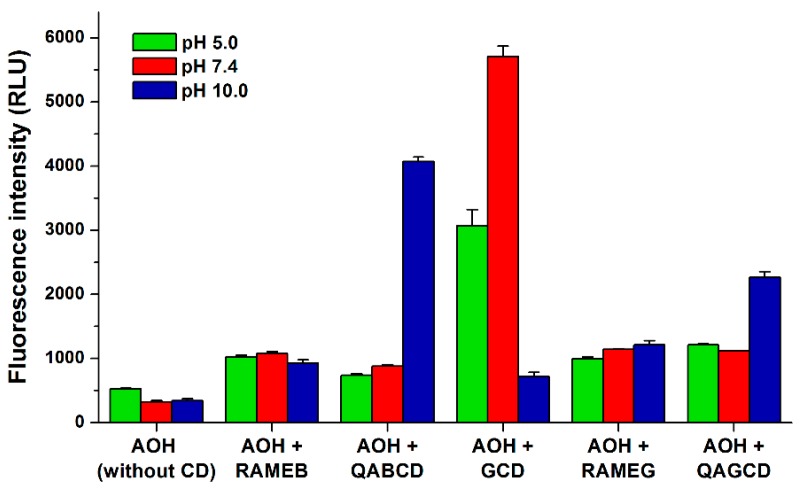
Absolute fluorescence signal (±SEM) of AOH (5 μM) in the absence and presence of CDs (10 mM each) in different buffers: sodium acetate (50 mM, pH 5.0), sodium phosphate (50 mM, pH 7.4), and sodium borate (50 mM, pH 10.0) (λ_ex_ = 345 nm, λ_ex_ = 460 nm; ex slit: 10 nm, em slit: 10 nm; RLU, relative light unit). Because of the lower aqueous solubility of BCD vs. other CDs tested, BCD has not been demonstrated here.

In a concentration-dependent fashion, each CD tested markedly increased the fluorescence emission signal of AOH (Figure 5; λ_ex_ = 345 nm, λ_em_ = 460 nm), except the native BCD, which only slightly enhanced the fluorescence of the mycotoxin (from 1.1- to 2.0-fold increase was observed in the presence of 5 μM AOH and 2.0 mM BCD). At pH 5.0 and 7.4, the native GCD proved to be the strongest fluorescence enhancer. However, at pH 10.0, quaternary ammonium derivatives were the most effective from this point of view, QABCD induced the strongest increase in the emission signal of AOH (Figure 5).

### 3.3. Binding Constants of Alternariol-Cyclodextrin Complexes

**Figure 6:**
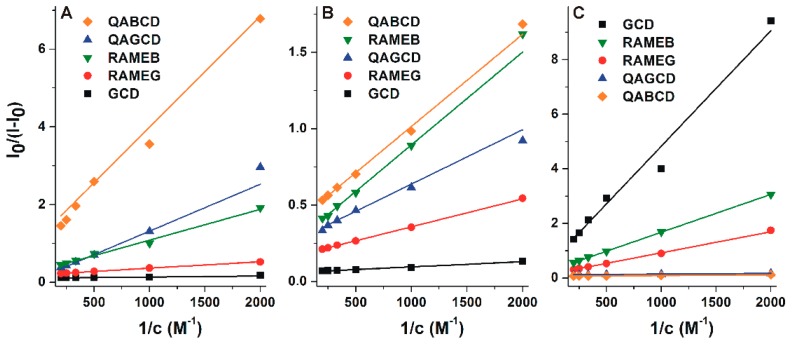
Benesi-Hildebrand plots (R^2^ = 0.95–0.99) of AOH-CD complexes in different buffers (λ_ex_ = 345 nm, λ_ex_ = 460 nm): (**A**) sodium acetate (50 mM, pH 5.0), (**B**) sodium phosphate (50 mM, pH 7.4), and (**C**) sodium borate (50 mM, pH 10.0).

Binding constants of AOH-CD complexes were determined based on fluorescence emission data employing the graphical application of the Benesi-Hildebrand equation (Equation (1); λ_ex_ = 345 nm, λ_em_ = 460 nm; log*K* values were also determined using other emission wavelengths to confirm our results). Benesi-Hildebrand plots showed good linearity (R^2^ = 0.95–0.99) with the 1:1 stoichiometry model (Figure 6).

The calculated log*K* values of AOH-CD complexes are listed in Table 1. Because of the lower aqueous solubility of BCD, its lower concentrations were applied (see Section 2.2). Therefore, the Benesi-Hildebrand plots of AOH-BCD complex are demonstrated separately in Figure S2. Log*K* values were in the range of 2.1–3.6, suggesting the strongly differing stabilities of AOH-CD complexes, depending on the CD applied and the environmental conditions (Table 1). Under acidic and physiological circumstances, the binding constants of RAMEB, GCD, and RAMEG complexes were significantly higher than at pH 10.0. At pH 5.0 and 7.4, the native GCD formed highly the most stable complexes with the mycotoxin. However, under alkaline circumstances (pH 10.0), AOH bound to the quaternary ammonium derivatives (QABCD and QAGCD) with approximately 10-fold higher affinity than to other CDs tested.

Since a marked emission peak also appeared around 410 nm at pH 5.0 regarding each CD examined (see in Figure 4A), the binding constants were also determined at pH 5.0 using 345 and 410 nm excitation and emission wavelengths, respectively. The calculated binding constants were same as the data determined applying 460 nm emission wavelength (see in Table 1).

To confirm the binding constants and stoichiometry determined based on the Benesi-Hildebrand equation, some data were also evaluated using the graphical application of the Scatchard equation (Equation (2); λ_ex_ = 345 nm, λ_em_ = 460 nm). The Scatchard plot of AOH-GCD complex (at pH 7.4) is demonstrated in Figure S3. This model also showed an excellent correlation with the 1:1 stoichiometry of complex formation, and gave similar binding constants to the evaluation performed with the Benesi-Hildebrand plot (e.g., the log*K* values regarding AOH-GCD complex were 3.21 ± 0.01 vs. 3.31 ± 0.05 based on the Benesi-Hildebrand plot and the Scatchard plot, respectively).

### 3.4. Modeling Studies

**Figure 7:**
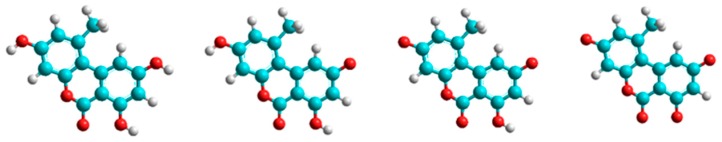
The energetically most favorable deprotonation route of AOH (from left to right: nonionic AOH, monoanionic AOH, dianionic AOH, and trianionic AOH) determined at AM1 method in the present work (C, O, and H atoms are indicated with blue, red, and white spheres, respectively). These results are in agreement with the previous study of Tu and co-workers (calculated at B3LYP using 6-311++G** basis set) [43].

**Figure 8:**
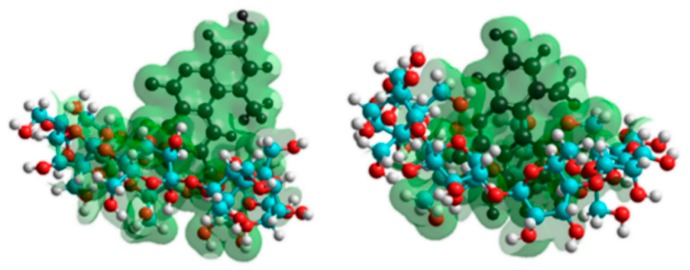
Equilibrium conformation with isosurface charge grids of AOH-BCD (**left**) and AOH-GCD (**right**) complexes. C, O, and H atoms of BCD and GCD are indicated with blue, red, and white spheres, respectively; while AOH is represented by black spheres.

To get a deeper insight into the complex formation, the AOH guest molecule and its deprotonated derivatives with the CD host molecules were investigated. The host molecules were represented by variation of the total molecular charge of the parent BCD and GCD molecules, while the deprotonated guest molecules were represented by their most stable conformers. Accordingly, the most probable deprotonation route of AOH is determined considering the formation energies associated to each deprotonation step. The differently protonated derivatives of AOH (Figure 7) were then used to investigate the complex formation. The inclusion of AOH by the cavities of BCD and GCD is demonstrated in Figure 8. A steric hindrance appeared regarding AOH-BCD interaction, so the mycotoxin is therefore able to sink deeper into the cavity of GCD.

Based on modeling studies, the log*K* values associated to the formation of AOH-CD complexes were calculated and are summarized in Table 2. These results support the higher stability of AOH-GCD vs. AOH-BCD complexes and are in agreement with the experimental data (Table 1) according to the tendencies of the protonation state or the electron density of the host cavity. Native and randomly methylated CDs form more stable complexes with the nonionic form of the mycotoxin, while the positively charged quaternary ammonium derivatives prefer the deprotonated forms of AOH.

### 3.5. Extraction of Alternariol from Aqueous Solution by Insoluble β-Cyclodextrin Bead Polymer

**Figure 9:**
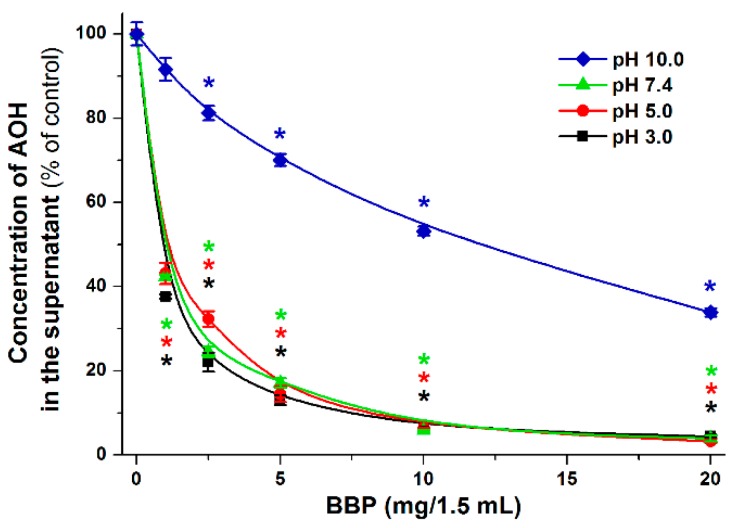
Extraction of AOH from aqueous solutions by insoluble β-cyclodextrin bead polymer (BBP). Effects of increasing concentrations of BBP (0.0, 1.0, 2.5, 5.0, 10.0, and 20.0 mg/1.5 mL) on the AOH (initial concentration: 2 μM) content of different buffers (50 mM sodium phosphate, pH 3.0; 50 mM sodium acetate, pH 5.0; 50 mM sodium phosphate, pH 7.4; and 50 mM sodium borate, pH 10.0). Incubations were carried out in a thermomixer (1000 rpm, 30 min, 25 °C; * *p* < 0.01).

Since the first acid dissociation constant (pK_a_) value of AOH is approximately 8.4 [44], we did not see reasonable to use lower pH than 5.0 in the previous experiments. However, AOH commonly appears in more acidic drinks (e.g., wine and tomato juice). Therefore, the removal of AOH by BBP was tested in the pH range of 3.0–10.0. The standard concentration of AOH (2 μM = 516.5 μg/L, in 1.5 mL buffer) was incubated in the presence of increasing amounts of BBP (1.0–20.0 mg/1.5 mL; see further details in Section 2.4), then the mycotoxin content of the supernate was quantified by HPLC-FLD (see Section 2.5). In a concentration-dependent fashion, BBP removed significant amounts of AOH from aqueous solutions (Figure 9). Insoluble β-cyclodextrin bead polymer was similarly effective mycotoxin binder in the 3.0-7.4 pH range. However, its AOH binding ability was strongly decreased at pH 10.0 compared to the other buffers applied. Under acidic and weakly alkaline circumstances (pH 3.0–7.4), even low amounts of BBP (1.0 mg/1.5 mL) caused approximately 60% decrease in the mycotoxin content of the solutions, and 10.0 mg/1.5 mL BBP almost completely removed AOH (Figure 9). However, at pH 10.0, only 47 and 66% decrease in the AOH content of the supernatant was observed in the presence of 10.0 and 20.0 mg/1.5 mL BBP, respectively.

**Figure 10:**
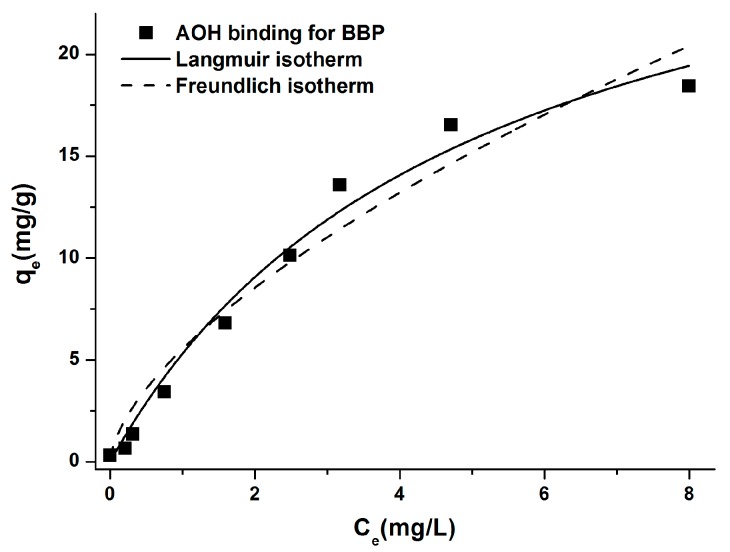
Langmuir (solid line) and Freundlich (dashed line) isotherms for the AOH binding of BBP in sodium phosphate buffer (50 mM, pH 3.0). The amount of bound AOH (mg) by BBP (g) and the amount of unbound AOH (mg) in the solution at equilibrium are indicated by *q_e_* and *C_e_*, respectively.

To quantitatively characterize the AOH binding ability of BBP, increasing AOH concentrations (0.2–15 μM in 1.5 mL buffer) were added to standard amount of BBP (2.5 mg) in sodium phosphate buffer (pH 3.0). Figure 10 demonstrates the corresponding Langmuir and Freundlich isotherms. Experimental data showed good fitting with both Langmuir (Equation (4)) and Freundlich (Equation (5)) models (R^2^ = 0.98 and 0.97, respectively). The Langmuir equilibrium constant (*K_L_*) was 0.16 ± 0.04 L/mg, and the maximum quantity of AOH (mg) bound per gram of BBP (*Q_0_* value) was 41.92 ± 3.94 mg/g. Furthermore, the Freundlich constant (*K_F_*) was 5.52 ± 1.31 (mg/g) × (L/mg)^1/n^, while the 1/n value was 0.74 ± 0.04.

### 3.6. Comparison of the Interactions of Alternariol with Native β-Cyclodextrin, Soluble β-Cyclodextrin Polymer, and Insoluble β-Cyclodextrin Bead Polymer

**Figure 11:**
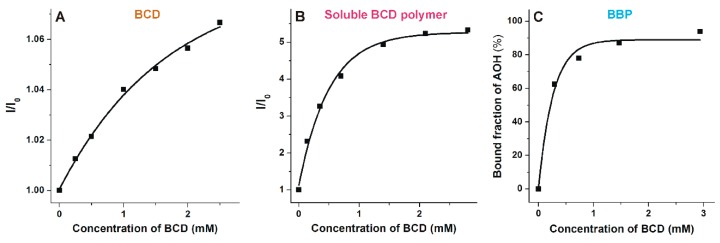
Comparison of the interactions of AOH with BCD, soluble BCD polymer, and BBP. Relative increase (I/I_0_) in the fluorescence emission intensity of AOH in the presence of increasing concentrations of BCD (**A**; λ_ex_ = 345 nm, λ_ex_ = 460 nm). Relative increase (I/I_0_) in the fluorescence emission intensity of AOH in the presence of increasing concentrations of soluble BCD polymer (**B**; λ_ex_ = 345 nm, λ_ex_ = 460 nm; concentration of BCD represents the molar BCD content of the applied amount of the soluble polymer). Bound fraction of AOH (% of total AOH concentration; calculated based on the data demonstrated in Figure 9) in the presence of increasing concentrations of BBP (**C**; concentration of BCD represents the molar BCD content of the applied amount of BBP). Each experiment was performed with 2 μM AOH concentration in sodium phosphate buffer (50 mM, pH 3.0).

Despite the stability of AOH-BCD complex is relatively low (Table 1), BBP very effectively decreased the mycotoxin content of aqueous solution (Figure 9). To test the hypothesis that BCD polymers may have considerably higher AOH binding capacity compared to the native BCD, the AOH binding ability of BCD, soluble BCD polymer, and BBP were compared in the same buffer (sodium phosphate, pH 3.0). Increasing amounts of BCD and soluble BCD polymer were added to AOH (2 μM), and then fluorescence emission spectra were recorded. According to the manufacturer’s description, the soluble BCD polymer and the BBP contains approximately 70 and 50 m/m% BCD, respectively. Based on these data, BCD concentrations contained by the applied amounts of soluble BCD polymer and BBP were calculated. The relative increase in the fluorescence emission signal of AOH (I/I_0_) in the presence of BCD and soluble BCD polymer was plotted as a function of the BCD concentration (Figure 11A,B). Similarly, the bound fraction of AOH in the presence of BBP (calculated based on the data represented in Figure 9) was also plotted vs. the BCD concentration contained by BBP (Figure 11C). Under the applied conditions, BCD induced less than 10% increase in the emission signal of AOH, while soluble BCD polymer caused more than five-fold enhancement in the fluorescence of the mycotoxin (Figure 11A,B). β-cyclodextrin polymers which are equivalent to 1.5–2.0 mM BCD content were able to bind close the total amount of AOH molecules (Figure 11A,B). However, the curve of BCD was far from the saturation even in the presence of 2.5 mM BCD (Figure 11A). Binding constants were quantified using the Benesi-Hildebrand equation (Equation (1); for BCD and soluble BCD polymer) and based on the free and bound fractions of AOH and BBP (Equation (7); for BBP), assuming 1:1 stoichiometry of complex formation (see details in Section 2.6). LogK values were 2.40 (±0.07), 3.40 (±0.01), and 3.67 (±0.07) regarding BCD, soluble BCD polymer, and BBP, respectively.

## 4. Discussion

AOH possesses three phenolic hydroxyl groups (Figure 1). Considering the previously reported pK_a_ values of AOH (8.4, 9.7, and 13.6) [44], it appears dominantly in its nonionic form (each phenolic hydroxyl group of the mycotoxin is protonated) at pH 5.0. With the elevation of the pH, significant changes in the fluorescence excitation and emission spectra of AOH were observed (Figure 2 and Figure S1), likely due to the deprotonation of the mycotoxin. In the applied pH range, the nonionic, monoanionic, and dianionic forms of the mycotoxin appear at relevant concentrations. Unfortunately, we cannot see separately the fluorescence spectra of the ionized forms because both monoanionic and dianionic AOH gradually appear with the elevation of the pH. Two distinct peaks were noticed in the excitation spectra of AOH (approximately at 350 and 410 nm; see in Figure 2A,B). The lack of the second peak at pH 5.0 and its appearance at higher pH values (pH 7.4 and 10.0) suggest that the higher excitation peak (at 410 nm) belongs to the ionized forms of the mycotoxin. This hypothesis is also supported by the emission spectra recorded using 410 nm excitation wavelength: no significant emission was noticed at pH 5.0, while an increasing emission peak appeared at higher pH values applied (Figure S1A). Furthermore, with the elevation of the pH, the first excitation peak (around 350 nm) decreased but did not disappear (Figure 2A,B). Thus, the first excitation peak can also be applied during the investigation of the ionized mycotoxins (at least monoanionic AOH). This hypothesis is also supported by the observation that significant emission signal of the mycotoxin was noticed using 345 nm excitation wavelength, even at pH 10.0 (Figure 2C).

Using 345 nm excitation wavelength, the emission spectrum of AOH seems to be one wide peak (Figure 2C). However, in the presence of CDs, we can distinguish two emission peaks (around 410 and 460 nm) at pH 5.0 and 7.4 (Figure 3 and Figure 4). Since AOH has tautomers [43], these two peaks likely belong to the two tautomeric forms of nonionic AOH. The tautomers can be visibly distinguished only in the presence of CDs, as it has also been reported regarding flavonoids fisetin and geraldol [45]. Log*K* values of AOH-CD complexes were determined at pH 5.0 using both 410 and 460 nm emission wavelengths, during which the calculated binding constants were identical. Based on these data, the tautomers of nonionic AOH bind to the same CD with the same affinity. With the elevation of the pH, the first emission peak became less dominant and/or disappeared at pH 10.0. Cationic CDs tested (QABCD and QAGCD) induced the strong decrease of the emission peak at 410 nm even at pH 7.4 (Figure 4), while it was not observed with other CDs (BCD, RAMEB, GCD, and RAMEG) (Figure 3). These observations support that the emission peak at 410 nm belongs to the nonionic AOH. Since quaternary ammonium derivatives form more stable complexes with the ionized form(s) of AOH (Table 1 and Table 2), QABCD and QAGCD can shift the chemical equilibrium toward the deprotonation of the mycotoxin, which consequently leads to the decreased emission peak at 410 nm.

GCD and QABCD induced a more than ten-fold increase in the fluorescence of AOH at pH 7.4 and 10.0, respectively. Cyclodextrins have been widely used to increase the performance and sensitivity of analytical techniques, including mycotoxin analysis [32,33,46]. Previous studies demonstrated that the fluorescence intensity of fluorescent mycotoxins can be strongly increased as a result of the formation of mycotoxin–CD host–guest type complexes [29,33,42,47]. It can be explained by the fact that water molecules can significantly quench the fluorescence of aromatic fluorophores [48]. Since the inclusion of the mycotoxin by the apolar CD cavity disrupts the hydration shell of the mycotoxin, the quenching effect of water molecules decreases, and consequently the increased fluorescence signal of AOH can be observed [27,41,42]. As the modeling studies suggest, non-ionized AOH sinks deeper into the GCD than into the BCD cavity (Figure 8). Therefore, the greater part of the fluorophore is protected from the external quencher molecules, which may explain the stronger enhancement in the fluorescence of AOH in the presence of GCD vs. BCD. Furthermore, at pH 10.0, the electrostatic interaction between the anionic form(s) of AOH and the positively charged quaternary ammonium CDs can increase the steric hindrance of AOH [49], leading to the strong increase in the fluorescence of the mycotoxin.

The stability of AOH-CD complexes was highly influenced by the environmental pH and by the CD applied (Table 1). At pH 10.0, QAGCD formed the most stable complex with the mycotoxin, while at pH 5.0 and 7.4, GCD formed the most stable complexes with the mycotoxin. The interaction of AOH with BCD was poorly affected by the pH. The binding constants of AOH-RAMEB, AOH-RAMEG, and AOH-GCD complexes were significantly higher at pH 5.0 and 7.4 (three-, six-, and 10-fold, respectively) vs. at pH 10.0. However, the stability of AOH-QABCD (three-fold at pH 7.4 and 10-fold at pH 10.0 compared to at pH 5.0) and AOH-QAGCD (seven-fold at pH 7.4 and 30-fold at pH 10.0 compared to at pH 5.0) complexes gradually increased with the elevation of the pH. Since at pH 10.0 the monoanionic and dianionic forms of AOH are dominant, these observations suggest that uncharged CDs generally prefer the nonionic form of AOH, while quaternary ammonium derivatives bind to the anionic form(s) of the mycotoxin with much higher affinity. Because QABCD and QAGCD are positively charged tetraalkylammonium cations, it is reasonable to hypothesize the ionic interaction between these CDs and AOH anion(s). Besides the inclusion, an electrostatic interaction contributes to the strong binding between the deprotonated AOH and the positively charged cavity entrance of γ-CD torus. It seems a close structural and functional analogy to rocuronium–Sugammadex interaction, where the aminosteroid non-depolarizing muscle relaxant guest binds with high affinity to the anionic γ-CD derivative [50]. The deprotonation of the AOH causes its negatively charged state, which is enhanced by the elevating pH. Modeling studies highlighted that this charge interacts with the host cavity and the electron density of the host molecule moderates the secondary host–guest interactions by the coulomb repulsion (RAMEB, RAMEG) or coulomb attraction (QABCD, QAGCD). As a result, the stability of AOH-QABCD and AOH-QAGCD complexes is increased by the deprotonation of AOH. In contrast, the opposite tendency was observed with AOH-RAMEB and AOH-RAMEG complexes. The slightly electron rich character of the GCD cavity causes reduced stability at higher deprotonated state of AOH. Overall, higher complex stabilities were observed with γ-CDs than with β-CDs in both spectroscopic (Table 1) and modeling (Table 2) studies, which is probably resulted from the steric hindrance appeared during the interactions with the smaller BCD cavity. Therefore, the entry of AOH molecule into the host cavity is deeper during the formation of AOH-GCD vs. AOH-BCD complexes (Figure 8).

Data showed good fitting to the 1:1 stoichiometry models regarding both Benesi-Hildebrand (Figure 6) and Scatchard plots (Figure S3), suggesting the 1:1 stoichiometry of complex formation. Furthermore, modeling studies also predicted the formation of 1:1 complexes. Despite the fact that GCD has a large cavity size (which theoretically makes possible the inclusion of more than one guest molecules), the deep inclusion of nonionic AOH inhibits the accommodation of another guest molecule due to the steric hindering. In each other case, when AOH is presented in its anionic form(s), the repulsive interaction between AOH molecules also prevents the formation of AOH-GCD complexes with higher stoichiometry.

The occurrence of mycotoxins in foods, feeds, and beverages is a considerable health and economic hazard worldwide. To reduce the mycotoxin content of these products, decontamination strategies are extensively studied, including filtration, heat treatment, ultraviolet radiation, addition of chemical reagents or adsorbents, etc. [51,52,53,54]. Insoluble CD polymers have been tested in few studies for decontamination/extraction purposes: they were successfully applied for the removal of patulin from apple juice [34,35], zearalenone from corn beer [37], and ochratoxin A from red wine [36]. Furthermore, CDs can also entrap the masked mycotoxin zearalenone-14-*O*-β-D-glucoside. BBP significantly reduced the zearalenone-14-*O*-β-D-glucoside content of aqueous solution [55]. Despite AOH-BCD complex showed similar binding constant in the pH range tested (pH 3.0–10.0; see in Section 3.3 and Section 3.6), BBP proved to be a less effective mycotoxin binder at pH 10.0 compared to other conditions examined (pH 3.0–7.4). The extraction of zearalenone by BBP was examined in our previous study under similar experimental conditions. AOH and zearalenone were removed from aqueous solutions by BBP with similar efficiency [37]. The successful extraction of AOH by BBP under acidic circumstances suggests the potential suitability of insoluble CD polymers regarding the decontamination of acidic beverages like wines (pH ~ 3.1–3.9 [56]) and tomato juice (pH ~ 4.0–4.2 [57]), beverages that are frequently contaminated with *Alternaria* mycotoxins [9,10].

The sorption isotherms can quantitatively describe the mycotoxin binding ability of BBP. Langmuir isotherm describes strictly homogenous monolayer adsorption, while Freundlich model does not have this restriction [58]. The Langmuir equilibrium constants of the interactions of AOH and zearalenone with BBP were in the same range (0.16 and 0.6 L/mg, respectively), while *Q_0_* value (describing the maximum quantity of the mycotoxin bound per gram of BBP) was significantly higher for AOH (42 vs. 3 mg/g) [37]. The Freundlich constant suggests the higher adsorptive capacity of BBP regarding AOH compared to zearalenone (5.5 and 1.2 (mg/g) × (L/mg)^1/n^, respectively) [37]. The heterogeneity index (*n*) for AOH-BBP interaction is close to 1, suggesting the relatively homogenous sorption of AOH by BBP.

The observation that AOH was removed from aqueous solutions by BBP with similar efficiency to zearalenone [37] was unexpected because AOH binds to BCD with considerably lower affinity compared to zearalenone (the log*K* values of AOH-BCD and zearalenone-BCD complexes are 2.2 and 4.0 at pH 5.0, respectively) [59]. As it is described in Section 3.6, both soluble BCD polymer and BBP were able to bind AOH with considerably higher efficiency than it was expected considering their BCD content. The calculated log*K* values listed in Section 3.6 do not describe precisely the interaction of AOH with CD polymers (because their BCD content was considered as BCD monomers). Nevertheless, these data clearly demonstrate the higher binding capacity of BCD polymers vs. the BCD monomer. The complexing potency of epichlorohydrin cross-linked BCD polymers sometimes surpasses that of the corresponding monomer (BCD) [60,61,62,63]. This phenomenon resulted from the cooperative/synergistic interaction of neighboring CD units, leading to the improved entrapment/complexing properties of the covalently linked rings [60,63]. Moreover, cross-linked CDs can be considered as partially and statistically dihydroxypropylated CD ethers (Figure S4), which show improved water solubility at room temperature (the disturbed H-bond system on the secondary face of the cavity entrance commonly enhances the aqueous solubility). The better solubility of BCD polymers (vs. BCD) and the cooperating lipophilic cavities, as well as the flexibly cross-linked CD network, support the more effective encapsulation and the more stable interaction with poorly water-soluble bulky guest molecules, such as AOH.

## 5. Conclusions

In summary, the interaction of mycotoxin AOH was examined with CDs and CD polymers. Most of the CDs induced a strong increase in the fluorescence signal of AOH. The native γ-CD proved to be the most successful fluorescence enhancer among the β- and γ-CDs tested, resulting in approximately 15-fold increase in the emission signal of AOH at pH 7.4. Native and methyl-substituted CDs formed more stable complexes with the nonionic form of the mycotoxin (under acidic and physiological conditions), while the quaternary ammonium derivatives prefer the ionized form(s) of AOH (which appear under alkaline circumstances). BBP successfully removed AOH from aqueous solutions under acidic and close to neutral conditions, but it was a less effective mycotoxin binder at pH 10.0. The AOH binding ability of soluble BCD polymer and BBP was significantly higher than expected based on their BCD content, suggesting the cooperative/synergistic interaction of neighboring CD units in the polymers applied. Based on the above-listed observations, CD technology seems a promising tool to improve the sensitivity of the fluorescence detection of AOH. Furthermore, CD polymers may be suitable for the development of new mycotoxin binders to remove AOH from contaminated beverages and consequently to decrease mycotoxin exposure.

## Figures and Tables

**Table 1:** Decimal logarithmic values of the binding constants (*K*; unit: L/mol) of AOH-CD complexes.

Mycotoxin-CD Complex	log*K* (±SEM)		
	pH 5.0	pH 7.4	pH 10.0
AOH-BCD	2.23 ± 0.01	2.52 ± 0.01	2.22 ± 0.03
AOH-RAMEB	2.50 ± 0.02	2.64 ± 0.01	2.11 ± 0.03
AOH-QABCD	2.38 ± 0.03	2.85 ± 0.04	3.40 ± 0.01
AOH-GCD	3.18 ± 0.06	3.21 ± 0.01	2.17 ± 0.03
AOH-RAMEG	2.96 ± 0.03	3.03 ± 0.03	2.23 ± 0.03
AOH-QAGCD	2.11 ± 0.03	2.97 ± 0.01	3.58 ± 0.03

Buffers used: sodium acetate (50 mM, pH 5.0), sodium phosphate (50 mM, pH 7.4), and sodium borate (50 mM, pH 10.0). See further details in Section 2.2 and Figure 6.

**Table 2:** Log*K* values (the unit of *K* is L/mol) of AOH-CD complexes based on theoretical calculations. Semi-empirical AM1 method with TIP3P solvation model was applied.

Host Molecule	Host Simulated as	Log*K* Values of AOH and Its Deprotonated Forms			
		AOH (Nonionic)	AOH (Monoanionic)	AOH (Dianionic)	AOH (Trianionic)
BCD	0 BCD	2.61	2.51	2.46	2.42
RAMEB	−1 BCD	2.52	2.41	2.32	2.14
QABCD	+1 BCD	2.48	2.81	2.98	3.40
GCD	0 GCD	3.17	3.11	3.04	2.61
RAMEG	−1 GCD	3.11	2.87	2.72	2.46
QAGCD	+1 GCD	2.29	2.88	3.12	3.46

The methyl derivatives (RAMEB and RAMEG), due to the electron releasing property of methyl substituent, were considered as negatively charged (−1) CDs. The quaternary ammonium derivatives (QABCD and QAGCD), because of the electron withdrawing character of the tetraalkylammonium moiety, were considered as the positively charged (+1) CDs.

## Supplementary Materials

The following are available online at https://www.mdpi.com/2218-273X/9/9/428/s1, Figure S1: Emission spectra of AOH using 410 nm excitation wavelength, Figure S2: Investigation of AOH-BCD complex formation based on the Benesi-Hildebrand equation (Equation (1)). Figure S3: Investigation of AOH-GCD complex formation based on the Scatchard equation (Equation (2)). Figure S4: Schematic representation of the chemical structure of epichlorohydrin cross-linked BCD polymer.
