# Supplementary material for: Interactions of Mycotoxin Alternariol with Cyclodextrins and Its Removal from Aqueous Solution by Beta-Cyclodextrin Bead Polymer
Source: Biomolecules. 2019 Aug 30;9(9):428. doi: 10.3390/biom9090428 (PMC6769471; doi:10.3390/biom9090428)
Supplement: Supplementary file 1 [file biomolecules-09-00428-s001.pdf]

biomolecules

MDPI

SUPPLEMENTARY MATERIALS

Article

Interactions of Mycotoxin Alternariol with Cyclodextrins and its Removal from Aqueous Solution by Beta-Cyclodextrin Bead Polymer

Eszter Fliszár-Nyúl [1,2], Beáta Lemli [2,3], Sándor Kunsági-Máté [2,3], Lajos Szente [4], and Miklós Poór [1,2,*]

[1] Department of Pharmacology, Faculty of Pharmacy, University of Pécs, H-7642 Pécs, Hungary
[2] János Szentágothai Research Centre, University of Pécs, H-7642 Pécs, Hungary
[3] Institute of Organic and Medicinal Chemistry, Medical School, University of Pécs, H-7624 Pécs, Hungary
[4] Cyclolab Cyclodextrin Research & Development Laboratory, Ltd., H-1097 Budapest, Hungary
* Correspondence: poor.miklos@pte.hu (M.P.)

Received: 31 July 2019; Accepted: 27 August 2019; Published: date

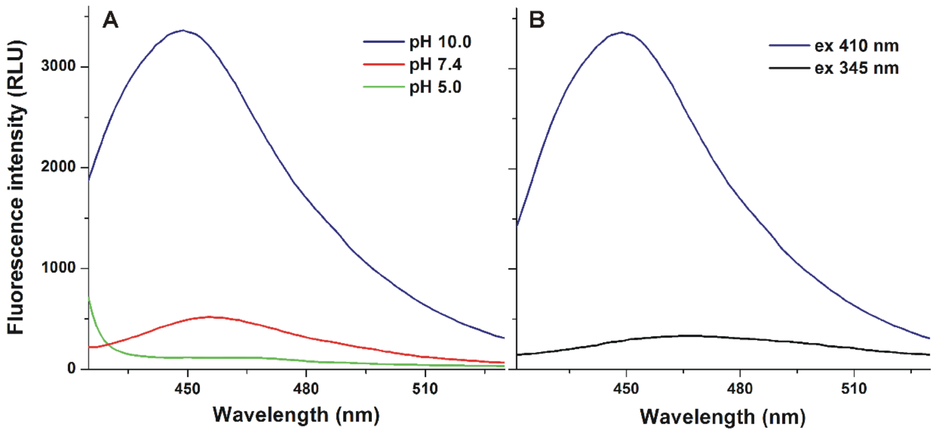

Figure S1. Emission spectra of AOH using 410 nm excitation wavelength. Fluorescence emission (**A**: λ_{ex} = 410 nm) spectra of AOH (50 μM) in in sodium acetate (50 mM, pH 5.0), sodium phosphate (50 mM, pH 7.4), and sodium borate (50 mM, pH 10.0) buffers. Fluorescence emission spectra of AOH (50 μM) in sodium borate buffer (50 mM, pH 10.0) using 345 (black line) and 410 nm (blue line) excitation wavelengths (**B**) (ex slit: 5 nm, em slit: 10 nm; RLU, relative light unit).

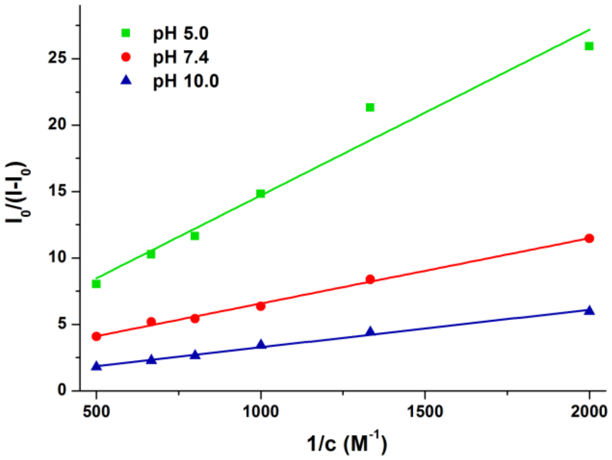

Figure S2. Investigation of AOH-BCD complex formation based on the Benesi-Hildebrand equation (Equation 1). Benesi-Hildebrand plots (R^2 = 0.96-0.99) of AOH-BCD complexes in sodium acetate (50 mM, pH 5.0), sodium phosphate (50 mM, pH 7.4), and sodium borate (50 mM, pH 10.0) buffers (λ_{ex} = 345 nm, λ_{ex} = 460 nm).

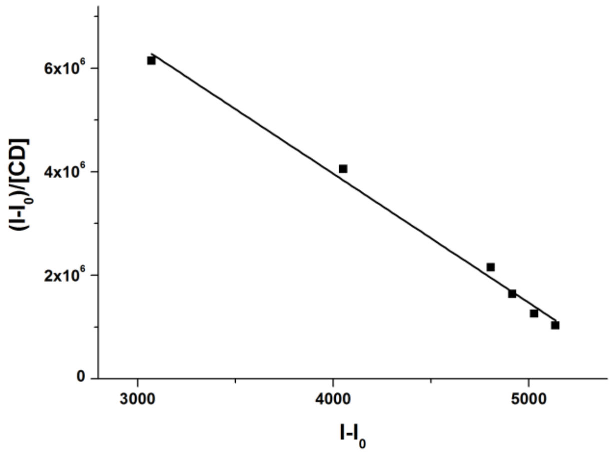

Figure S3. Investigation of AOH-GCD complex formation based on the Scatchard equation (Equation 2). Scatchard plot (R^2 = 0.99) of AOH-GCD complex in sodium phosphate (50 mM, pH 7.4) buffer (λ_{ex} = 345 nm, λ_{ex} = 460 nm; AOH: 5 µM; GCD: 0.0, 0.5, 1.0, 2.0, 3.0, 4.0, and 5.0 mM).

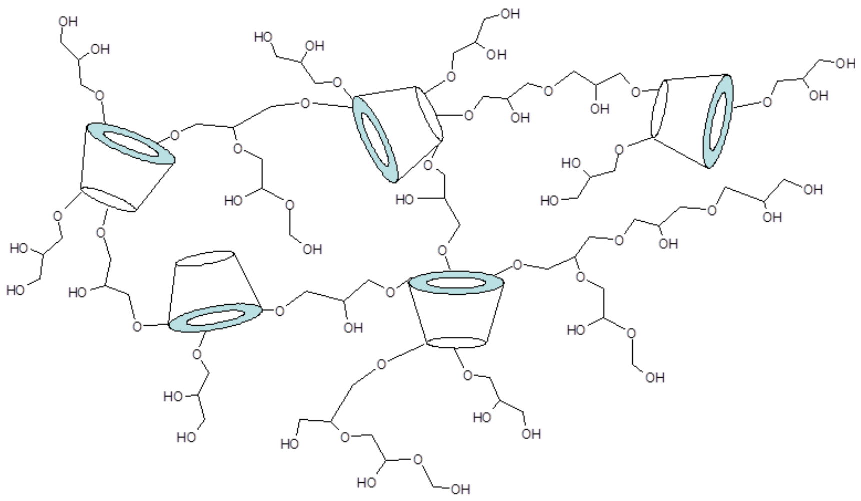

Figure S4. Schematic representation of the chemical structure of epichlorohydrin cross-linked BCD polymer.
